# Identification of A Gene Set Associated with Colorectal Cancer in Microarray Data Using The Entropy Method

**DOI:** 10.22074/cellj.2019.5688

**Published:** 2018-08-07

**Authors:** Fatemeh Bahreini, Ali Reza Soltanian

**Affiliations:** 1 Department of Molecular Medicine and Genetics, School of Medicine, Hamadan University of Medical Sciences, Hamadan, Iran; 2 Modeling of Noncommunicable Diseases Research Center, School of Public Health, Hamadan University of Medical Sciences, Hamadan, Iran; 3 Department of Biostatistics and Epidemiology, School of Public Health, Hamadan University of Medical Sciences, Hamadan, Iran

**Keywords:** Cancer, Colorectal, Microarray, Statistical Model

## Abstract

**Objective:**

We sought to apply Shannon’s entropy to determine colorectal cancer genes in a microarray dataset.

**Materials and Methods:**

In the retrospective study, 36 samples were analysed, 18 colorectal carcinoma and 18 paired normal tissue samples. After identification of the gene fold-changes, we used the entropy theory to identify an effective gene set. These genes were subsequently categorised into homogenous clusters.

**Results:**

We assessed 36 tissue samples. The entropy theory was used to select a set of 29 genes from 3128 genes that had fold-changes greater than one, which provided the most information on colorectal cancer. This study shows that all genes fall into a cluster, except for the R08183 gene.

**Conclusion:**

This study has identified several genes associated with colon cancer using the entropy method, which were not detected by custom methods. Therefore, we suggest that the entropy theory should be used to identify genes associated with cancers in a microarray dataset.

## Introduction

Cancer is one of the leading causes of death in both developed and developing countries. Increasing life expectancy will cause a worldwide increase in the cancer burden, especially in less developed countries (1). In 2012, 14.1 million new cancers were detected, with an estimated 8.2 million deaths from cancer worldwide (2).

Colorectal cancer is one of the most common types of cancer. Despite progress in screening and diagnostic methods, it is the third most common cancer in the world. In addition, it is ranked fourth and the fifth among cancers in developed and undeveloped countries, respectively. Worldwide, colon cancer is the third most frequent cancer in males and second most frequent in females. Colorectal cancer comprises 10% of all malignancies in males and 9.2% of total cancers in women. Approximately 55% of people with colorectal cancer live in developed countries (2). 

Colorectal cancer in the European countries has the highest incidence among malignancies and the second leading cause of death among malignant diseases in the countries (3). The latest studies show that the annual rate of colorectal cancer is increasing worldwide (2, 4). However, over 95% of colorectal cancer can be treated if detected early (5).

Therefore, early diagnosis of colorectal cancer and identification of cancer prognosis is very important. One of the prognostic factors for colorectal cancer is the gene set associated with this disease. We can use gene expression information extracted by microarray technology to determine the gene set associated with colorectal cancer. At the moment, microarray data has been used to determine the disease prognosis and the classification of genes associated with cancers. 

One of the debatable issues in analysis of microarray data is the selection of a range of genes associated with cancer due to the large number of genes examined compared to the number of cases in the microarray data. This may lead to bias in gene selection and classification (6). Therefore, to solve this problem, advanced mathematical methods can be used to reduce the number of genes (7).

Shannon’s entropy is one of the techniques for reducing the dimension of a large dataset such as microarray data that has recently been considered by researchers. Researchers use entropy to classify the genes into categories according to gene similarities and dissimilarities. The gene selection algorithm is again used to modify the selected gene list so that at least one subset gives the desired accuracy of the classification. The present study has used Shannon’s entropy method to determine up-regulated, overexpressed genes associated with colorectal cancer. These genes could potentially be used as a new therapeutic target. 

## Materials and Methods

### Dataset

In the retrospective study, we used data from a study by Notterman et al. (8) that evaluated 18 colon adenocarcinoma and 18 paired normal tissue samples obtained from the Cooperative Human Tissue Network (available at: http://genomics-pubs.princeton.edu/ oncology/Data/CarcinomaNormalDatasetCancerResearch. txt). A pathologist reviewed the adenocarcinoma samples. The patients had a mean ± SD age of 67.56 ± 14.09 years. Of all patients, 66.6% were female. 

### Shannon’s entropy 

The information from the text file provided by Notterman et al. (8) was exported to SPSS 16 version 16.0 for Windows (Inc., Chicago, IL). Then, we separately calculated the average of the gene expressions in the tumour and normal tissues. Next, we determined the fold-change (calculated formula) with respect to equation 1. 

[Equation 1] $$\mathrm{Fold change}=2^{\mathrm{|log2 (avg(C)/(avg(N)|}}$$

Where, ave(C) and ave(N) denote expression intensity levels of the tumour and normal tissues. 

We used Shannon’s entropy theory (equation 2) to select a gene set that affected colorectal cancer, which had the most mathematical information about colorectal cancer (9). 

[Equation 2] H(X)=-Σim=1p(xi)log p(xi)

In addition, the uncertainty of genes was measured by equation 3. In other words, the interdependency of two genes, X and Y, was defined as: 

[Equation 3] I(X,Y)=H(X)+H(Y)-H(X,Y)

In equation 3, H(X,Y), H(X), and H(Y) are the mutual information, the entropy of gene-X and gene-Y, respectively. The normalized mutual information, U(X,U), between the two genes (e.g., X and Y) was defined as: 

[Equation 4] 0≤U(X,Y)=2I(X,Y)(H(X)+H(Y))≤1

The values of one and zero for U(X,Y) denote that genes *X* and *Y* have a high mutual relevance (e.g., dependent) and low mutual relevance (e.g., independent), respectively.

If *S* is a collection of selected genes, the degree of suitability and complementarity of the genes are determined by equations [5] and [6], respectively.

[Equation 5] ŋ1=ΣiƐSU(g i,c)

[Equation 6] ŋ1=Σi, jƐSU(g i,g j)

where gi represents ith the gene and represents the corresponding cluster. Gene set S must be selected such that the gene relevance rate is maximized (equation 5), while the gene excess rate is minimized (equation 6).

### Data clustering

After selecting a set (S) of genes, which had the most information on colorectal cancer using the entropy technique, we applied a two-way hierarchical clustering method to categorise them into clusters. First, we put each gene in a vector to cluster the genes; second, we used the Euclidean distance to determine the distance between the genes (10). We used MATLAB (version 8) to determine a set, S as a collection of selected genes, the degree of suitability and complementarity of the genes is given in equations 5 and 6. In addition, the EntropyExplorer and Heatmap packages of R3.2.2 software were used to compute the entropy information genes and dendrogram drawings.

## Results

We assessed 36 tissue samples, 18 adenocarcinoma tissues and 18 paired normal samples. In the initial assessment of 7465 cDNAs and expression sequence tags (ESTs) available (http://genomics-pubs.princeton. edu/oncology), 3128 genes that had a fold-change greater than one were defined. In the second stage, we implemented the entropy theory and selected a set of 29 genes, which had the most mathematical information on colorectal cancer. Table 1 lists these genes.

Table 2 shows the gene name, aliases and locations. In addition, comparison between results of the study and previous studies was shown in Table 2.

**Fig.1:**
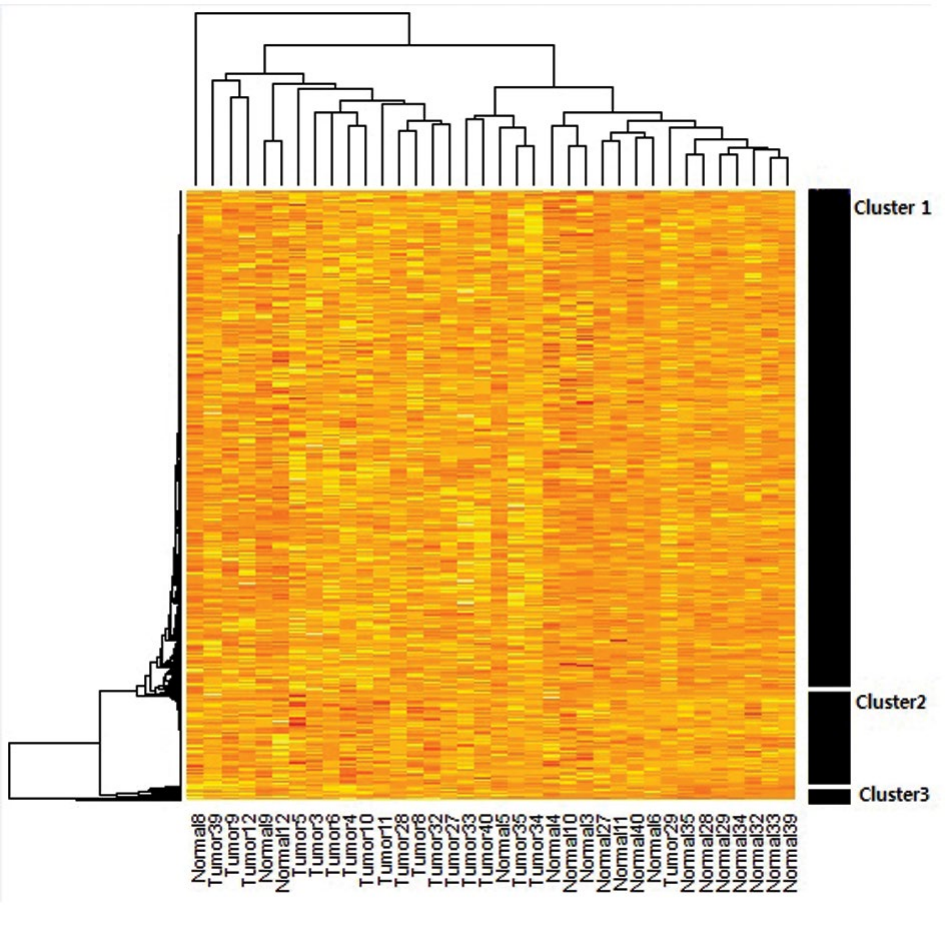
Cluster map derived from two-way cluster analysis by the hierarchical method. Approximately 3000 common genes in tumour tissues and paired normal tissues were combined in a matrix. Clustering was performed on this matrix. Each colour patch on the cluster map indicates the expression intensity level of the associated gene in that tumour and normal tissue samples. The colour patches on the cluster map have continuity on expression levels from yellow (highest) to red (lowest).

**Fig.2:**
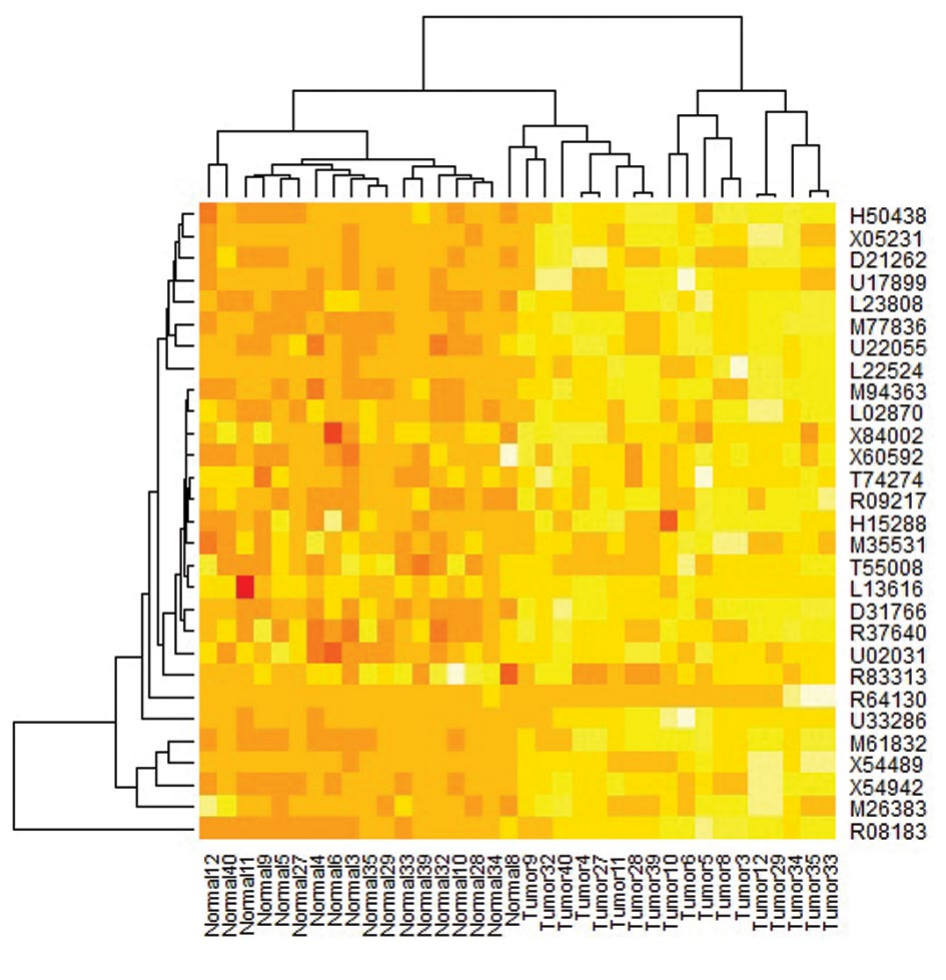
Cluster map derived from two-way cluster analysis with the hierarchical method. We combined 29 common genes in tumour and normal tissues in a matrix. Clustering was performed on this matrix. Each colour patch on the cluster map indicates the expression intensity level of the associated gene in that tumour and normal tissue samples. The colour patches on the cluster map have continuity on expression levels from yellow (highest) to red (lowest).

The dendograms of genes clustered are shown in Figures 1 and 2. Dendrogram 1 (Fig .1) is related to the clustering of all genes (n=3128), while dendrogram 2 (Fig .2) is based on 29 selected genes according to the entropy theory. For dendrograms 1 and 2, samples are shown along the horizontal axis (x) and the selected gene set is displayed along the vertical axis (y).

The cluster analysis on expression intensity of 3128 genes indicates that they can be divided into 3 clusters. In clusters 1 and 3 (Fig .1), it is clearly seen that expression intensity in normal tissue is more than tumour tissue (darker colour indicates greater expression), whereas expression intensity of normal samples in cluster 2 appears to be less than the tumour samples.

Therefore, in the final data track, genes were considered that had greater expression of tumour tissue compared to normal tissue, as shown by a fold- change greater than 1.

Although the primary cluster analysis divided the genes into 3 clusters, we did not discover any regular pattern. 

**Table 1:** A gene set defined on microarray data based on the entropy technique


Accession no.	Description	Intensity in tumour	Intensity in normal	Tumour/ normal

R37640	yf61b04.s1 Homo sapiens cDNA clone 26670 3′ similar to gb:M96995 GROWTH FACTOR RECEPTOR-BOUND PROTEIN 2 (HUMAN)	22.17	0.06	369.50
M94363	Human lamin B2 (LAMB2) gene and ppv1 gene sequence	15.89	0.11	144.45
L13616	Human focal adhesion kinase (FAK) mRNA, complete cds	6.94	0.06	115.67
X60592	Human CDw40 mRNA for nerve growth factor receptor-related B-lymphocyte activation molecule	14.67	0.17	86.29
H50438^*^	yo29f11.s1 Homo sapiens cDNA clone 179373 3′ similar to gb:S78187 M-PHASE INDUCER PHOSPHATASE 2 (HUMAN)	46.72	0.72	64.89
D31766	Human mRNA for KIAA0060 gene, complete cds	29.06	0.50	58.12
T55008	yb45h04.s1 Homo sapiens cDNA clone 74167 3′ similar to gb:X02619_rna1 APOLIPOPROTEIN A-II PRECURSOR (HUMAN)	115.70	2.22	52.12
H15288	ym30g12.s1 Homo sapiens cDNA clone 49810 3′	4.17	0.11	37.91
L22524	Human matrilysin gene, exon 6 and complete cds	79.22	2.67	29.67
M35531	Human GDP-L-fucose:beta-D-galactoside 2-alpha-l-fucosyltransferase mRNA, complete cds	12.61	0.44	28.66
L02870	"Human alpha-1 type VII collagen (COL7A1) mRNA, complete cds"	12.56	0.44	28.55
R64130	yi18h03.s1 Homo sapiens cDNA clone 139637 3′ similar to gb:M54995 PLATELET BASIC PROTEIN PRECURSOR (HUMAN)	68.89	2.50	27.56
X05231^*^	Human mRNA for collagenase (E.C. 3.4.24)	41.78	1.56	26.78
T74274	yc56h07.s1 Homo sapiens cDNA clone 84733 3′ similar to gb:X05199 PLASMINOGEN PRECURSOR (HUMAN)	4.39	0.17	25.82
U02031	Human sterol regulatory element binding protein-2 mRNA, complete cds	18.39	0.72	25.54
R09217	yf26b08.s1 Homo sapiens cDNA clone 127959 3′ similar to gb:X07173 INTER- ALPHA-TRYPSIN INHIBITOR COMPLEX COMPONENT II (HUMAN)	2.72	0.11	24.73
U22055^*^	Human 100 kDa coactivator mRNA, complete cds	72.98	3.89	18.76
X84002	Homo sapiens TAFII20 mRNA for transcription factor TFIID	5.72	0.33	17.33
X54489^*^	Human gene for melanoma growth stimulatory activity (MGSA)	105.06	9.00	11.67
M61832^*^	Human S-adenosylhomocysteine hydrolase (AHCY) mRNA, complete cds	123.06	20.61	5.97
M77836^*^	Human pyrroline 5-carboxylate reductase mRNA, complete cds	95.33	17.83	5.35
L23808^*^	Human metalloproteinase (HME) mRNA, complete cds	71.22	14.00	5.09
D21262^*^	Human mRNA for KIAA0035 gene, partial cds	55.56	10.89	5.10
R08183^*^	yf18e03.s1 Homo sapiens cDNA clone 127228 3′ similar to SP:CH10_BOVIN Q04984 10 KD HEAT SHOCK PROTEIN, MITOCHONDRIAL ;	439.50	91.17	4.82
U33286^*^	Human chromosome segregation gene homolog CAS mRNA, complete cds	98.67	21.33	4.63
R83313	yp82d03.s1 Homo sapiens cDNA clone 193925 3′ similar to gb:X63564 DNA- DIRECTED RNA POLYMERASE II LARGEST SUBUNIT (HUMAN)	260.39	59.94	4.34
M26383	Human monocyte-derived neutrophil-activating protein (MONAP) mRNA, complete cds	246.83	57.11	4.32
X54942^*^	Homo sapiens ckshs2 mRNA for Cks1 protein homologue	172.00	42.15	4.08
U17899^*^	Human chloride channel regulatory protein mRNA, complete cds	66.44	16.39	4.05


*; Custom genes in this study and the Notterman et al. (8) study.

**Table 2:** New genes identified using the entropy method in the present study


Accession no.	Gene name	Known as/aliases	Location	References

R37640	*GROWTH FACTOR RECEPTOR-BOUND PROTEIN 2*	Grb3-3, MST084, ASH, MSTP084, NCKAP2, EGFRBP-GRB2	17q25.1	Saucier and Rivard (11); Pabla et al. (12).
M94363	*Lamin B2 (LAMB2)*	LAMS, NPHS5	3p21.31	Brackenridge et al. (13)
L13616	*Focal adhesion kinase (FAK)*	PTK2 protein tyrosine kinase 2 (PTK2)	8q24.3	Golubovskaya et al. (14); Lark et al. (15)
X60592	*CDw40*	CD40, p50, Bp50, or TNFRSF5	20q13.12	Pang et al. (16); Zhou et al. (17)
D31766	*Glucosamine-6-phosplate deaminase (GNPDA)*	GPI, HLN, GNP1, GNPI, or GNPDA1	5q31.3	He et al. (18); Monjazeb et al. (19)
T55008	*APOLIPOPROTEIN A-II PRECURSOR*	apo(a)	11q23.3	Vargas et al. (20)
H15288	*Nuclear and coiled body phosphor protein 1 (NOLC1)*	P130, KIAA0035, NOPP140, NOPP130 or NS5ATP13	10q24.32	Xu et al. (21)
L22524	*Matrilysin gene*	MMP-7, MPSL1 or PUMP-1	11q22.2	Kumar et al. (22); Kioi et al. (23)
M35531	*GDP-L-fucose:beta-D-galactoside 2-alpha-l-fucosyltransferase*	FUT2, SE; Se2; sej; SEC2; B12QTL1, alpha (1,2) fucosyltransferase	19q13.33	Sun et al. (24)
L02870	*Alpha-1 type VII collagen (COL7A1)*	EBD1; EBR1; EBDCT; NDNC8	3p21.31	Chittenden et al. (25)
R64130	*PLATELET BASIC PROTEIN PRECURSOR*	PBP; TC1; TC2; TGB; LDGF; MDGF; TGB1; B-TG1; CTAP3; CXCL7; NAP-2; SCYB7; THBGB; LA-PF4; THBGB1; Beta-TG; CTAPIII; CTAP-III	4q13.3	Grepin et al. (26)
T74274	*PLASMINOGEN PRECURSOR*	Pg; AI649309	17 A1; 17 8.5 cM	de Bruin et al. (27); Didiasova et al. (28)
U02031	*Sterol regulatory element binding protein-2*	SREBP2, bHLHd2, SREBP-2	22q13.2	Babel et al. (29)
R09217	*ALPHA-TRYPSIN INHIBITOR COMPLEX COMPONENT II*	Inter-Alpha-Trypsin Inhibitor Heavy Chain 2, H2P; SHAP, ITI-HC2	10p14	Chen et al. (30)
X84002	*TAFII20*	TAF2J, TAF12	1p35.3	Ma et al. (31)
R83313	*DIRECTED RNA POLYMERASE II LARGEST SUBUNIT (POLR2A)*	RPB1; RPO2; POLR2; POLRA; RPBh1; RPOL2; RpIILS; hsRPB1; hRPB220	17p13.1	Lui et al. (7)
M26383	*Monocyte-derived neutrophil-activating protein (MONAP)*	CXCL8, IL8; NAF, GCP1, LECT, LUCT, NAP1, GCP-1, LYNAP, MDNCF, NAP-1	CXCL8	Yap et al. (32)


## Entropy analysis

After we selected 29 genes associated with colorectal cancer according to the entropy theory (Table 1), we attempted to cluster them in terms of gene expression intensity level in two directions, gene and tissue. The vertical axis of Figure 2 shows that all genes fall into a cluster, except for the R08183 gene.

## Discussion

The present study reported the application of the entropy theory to identify and select the most important gene set associated with colorectal cancer in a large dataset such as the microarray dataset. Also, we used a two-way hierarchical clustering algorithm approach to cluster the genes. 

The method used in our work, unlike conventional methods, considers the correlation between genes and uses the normalized mutual information (e.g., relevance and redundancy between genes). In this technique, the number of genes that contain colorectal cancer information increase and the number of unrelated genes (e.g., genes that give little notice to cancer) decrease. In many studies for gene clustering, the correlation between genes is not used; hence, the results may not be valid. In this study, we have taken into consideration the correlation between genes in their selection process. Under the current study, there were very few folding coding genes (transcripts) higher than 2 that agreed with the results reported by Notterman et al. (8). In analysing the microarray data, both the up-regulated and the down-regulated genes were important; however, we only assessed up-regulated genes in this study. 

Our study found 29 genes associated with colorectal cancer, which were more genes attributed to colorectal cancer compared to the Notterman et al. (8) study. The reason for this was to use the entropy theory in our study, whereas the previous study did not use normalized mutual information between the genes. A comparison of the results of our study with those reported by Notterman et al. (8) showed that both studies agreed with the discovery of 12 genes associated with colorectal cancer. However, the current study identified 17 genes associated with colorectal cancer, which were not identified in the Notterman et al. study. Their study confirmed 6 genes (KIAA0101, GRO-g, L-iditol-2 dehydrogenase, RNA POL II subunit, myoblast cell surface antigen 24.1 DS, and GTF3A) associated with colorectal cancer, which we did not identify. These genes do not have a large amount of fold-change. We identified genes that had a large fold-change in the current study. Of the 17 genes we discovered, 14 (82.35%) had a fold-change over 20. Therefore, it could be seen that the method used in this study more effectively discovered these genes compared to other studies.

In this study, we used cluster analysis to categorise 29 genes into 2 clusters. The first cluster included 28 genes and the second cluster contained only the R08183 gene. Liu et al. (7) did not observe this finding in their study. They detected 9 genes at the reduced final feature set, which was much lower than the number of genes identified in the current study. Our dendrogram showed that the difference between R08183 expressions in cancer tissue compared to normal tissue was much higher than other genes. This finding was not confirmed by Liu et al. (7). We included only the up-regulated genes in the model, whereas they included both up-regulated and down-regulated genes. Our study showed that 3 genes (R37640, M94363, and L13616) had fold-changes greater than 100, whereas Liu et al. (7) did not refer to any of these genes as colorectalrelated genes. Saucier and Rivard (11) and Pabla et al. (12) also showed an association of the R37640 gene with colorectal cancer. Brackenridge et al. (13) same our study reported a significant association between the M94363 gene and colorectal cancer. An association between the L13616 gene with colorectal cancer was confirmed by Golubovskaya et al. (14), Lark et al. (15), and in the current study. However, Liu et al. (7) and Notterman et al. (8) did not observe this association. Given the different results obtained, we propose to emphasize these genes in future studies. 

Recently, a method has been introduced based on the kernel function for selection and clustering of genes. We did not use this method due to the difficulty of choosing the kernel function (16).

## Conclusion

This study identified several genes associated with colon cancer by the entropy method, which have not been detected by custom methods. Therefore, we propose that researchers use the entropy method to identify genes associated with cancers in a microarray dataset.
